# Co-Intercalation of Sericite by Cationic and Anionic Surfactants and the Mechanical Properties of Sericite/Epoxy Resin Composites

**DOI:** 10.3390/ma18112486

**Published:** 2025-05-26

**Authors:** Yu Liang, Yajuan Xu, Yiman Jiang, Lingfeng Yu, Hao Ding

**Affiliations:** 1 School of Materials Science and Technology, Shenyang University of Chemical Technology, Shenyang 110142, China; xyjqbyl@163.com (Y.X.); 13841270351@163.com (Y.J.); 2 Department of Mechanics and Aerospace Engineering, Southern University of Science and Technology, Shenzhen 518055, China; yulf2023@mail.sustech.edu.cn; 3 School of Materials Science and Technology, China University of Geosciences (Beijing), Beijing 100083, China

**Keywords:** sericite, cationic–anionic, intercalation, mechanism, mechanical properties

## Abstract

Although the intercalation of sericite with cationic surfactants has been extensively studied, successful intercalation using anionic surfactants has yet to be achieved. This article aims to partially or fully intercalate sericite with an anionic surfactant, and to develop the corresponding sericite/polymer nanocomposite. To achieve this goal, we modified raw sericite by thermal modification, acid activation, and sodium modification. The modified sericite was then co-intercalated by cationic surfactant hexadecyl trimethyl ammonium bromide (CTAB) and anionic surfactant sodium dodecyl sulfate (SDS). The intercalated sericite was characterized by XRD, FTIR, SEM, DTA-TG, and a contact angle tester. The optimized sample had a layer-to-layer distance of 6.56 nm and an intercalation rate of 95.7%. Compared with raw sericite, the new organo-sericite showed increased hydrophobicity. A proposed mechanism for the intercalation by these surfactants was also discussed. Finally, sericite/epoxy composite was prepared by using the new organo-sericite as the raw material, demonstrating significantly improved mechanical properties compared to pure epoxy resin (72% improved for bending strength and 62% improved for tensile strength, compared with pure epoxy resin). The new organo-sericite is a promising filler in epoxy resin to enhance thermal stability and mechanical performance of the composite.

## 1. Introduction

Sericite is a natural 2:1 type phyllosilicate [1] characterized by the relatively high negative charge of its aluminosilicate layers as a result of tetrahedral substitutions of Al^3+^ for Si^4+^ [2,3]. This negative charge is balanced by K^+^, which occupies the 12-coordinated interlayer space. Compared with other phyllosilicates, such as montmorillonite and vermiculite, sericite is seldom researched, because it is neither water-swellable nor ion-exchangeable [4]. This makes preparing sericite-based nanocomposite difficult. Compared with other phyllosilicates, however, sericite exhibits unique properties, such as a high aspect ratio [5], unique properties of shielding, and absorbing ultraviolet radiation [6], making it quite promising in new and growing applications among pigments [7], biomimetic material [8,9], sensors [10,11], and photoelectrodes [12,13], just to name a few. Among them, a sericite/polymer nanocomposite attracted much attention. Sericite/epoxy nanocomposites were first made in 2008 by a conventional thermoset process [14]. Following that, sericite/polymer nanocomposites with various polymer matrices, such as epoxy resin [15], polyamide [16], polylactic acid (PLA) [17], acrylonitrile-butadiene-styrene (ABS) [18], polyimide [19], and polystyrene [16,17,18,19,20] were created. However, compared with other phyllosilicates, sericite/epoxy nanocomposites were seldom researched, which was mainly due to the non-ion-exchangeable properties of sericite.

In order to prepare sericite/polymer nanocomposite, sericite needed to be modified first to change its surface properties from pristine hydrophilic to relative hydrophobic, ensuring its further compatibility with polymer matrices, which are typically hydrophobic. Therefore, much work has been conducted on the intercalation of sericite to facilitate its further usage in the relevant nanocomposites. Most of the related literature usually modified sericite first, in order to reduce the negative charge between the interlayers, improve cation exchange capacity, and allow cation exchanges, mainly by replacing pristine K^+^ in the interlayer with other cations of small molecular size, such as Na^+^ [2,21] and Li^+^ [22,23,24]. Then the modified sericite was intercalated by cationic surfactants to expand its interlayer and render it compatible with hydrophobic polymers. The widespread use of cationic surfactants can be attributed to their structure, which combines cations and alkyl chains as a whole. The cations facilitate exchange with the cations in the interlayer of sericite, while the alkyl chains effectively enlarge the interlayer space of sericite. Until now, the most chosen cationic surfactants were cetyltrimethylammonium bromide [2], octadecyltrimethylammonium bromide, dioctadecyldimethylammonium bromide [25], and hexadecyltrimethylammonium bromide [24]. Some of the cationic surfactants decompose around 180 °C, making them unsuitable for high-temperature processes [26]. Compared to most cationic surfactants, anionic surfactants offer more advantages, such as lower cost and better thermal properties, making them a favorable option for intercalating sericite [27,28]. Based on current information, no one has ever intercalated sericite with anionic surfactants or nonionic surfactants.

The intercalation of sericite with anionic surfactants remains a challenge. The objectives of this article were to fully or partially intercalate sericite with an anionic surfactant to create a new type of organo-sericite. The prepared organo-sericite was then used to prepare a sericite/epoxy nanocomposite. It was anticipated that the prepared sericite/epoxy nanocomposite would have better mechanical properties than pure epoxy resin. The new organo-sericite is expected to be used as a filler in polymer composites to improve mechanical properties, as well as in other applications such as oil well drilling and paints.

## 2. Materials and Methods

### 2.1. Materials

The characterization of sericite raw materials (S_0_) purchased from Anhui Province, China, was reported previously [4]. The purity of the raw sericite is 93.2%, with 6.8% of quartz included. The main compositions are SiO_2_ (45.71%), Al_2_O_3_ (28.32%), Fe_2_O_3_ (3.04%), K_2_O (8.09%), and MgO (2.12%). The loss on ignition (LOI) is 4.47%. The chemical formula of S_0_ is (K_0.79_Na_0.11_Ca_0.01_)(Al_1.64_Ti_0.02_Fe_0.18_Mg_0.24_)(Al_0.92_Si_3.08_)O_10_(OH)_2_. After structural modification by using methods and data stated in a previously published article [4]—namely thermal activation (at 800 °C for 1 h in air), acid treatment (95 °C, 4 h in 5 M nitric acid), and Na-exchange treatment (95 °C, 3 h in saturated sodium chloride solution)—it was used as a raw material in this study. After modification, the cation exchange capacity (CEC) was improved from 7.42 mmol/100 g (S_0_) to 56 mmol/100 g (S_3_, the sericite after three-step modification). The intercalation of sericite with hexadecyl trimethyl ammonium bromide (CTAB) followed the procedure mentioned in a previously published article [2]. The process involved adding S_3_ into the suspension of DMSO and DI water (volume ratio 92:8) with CTAB 15 times the CEC of sericite, dissolved at 80 °C for 12 h. This was followed by washing with DI water 5 times, centrifugating at 4500 rpm for 4 min, and drying at 50 °C for 12 h. All other reagents (such as nitric acid, sodium chloride, cetyltrimethylammonium bromide (CTAB), dimethyl sulfoxide (DMSO), and sodium dodecyl sulfate (SDS)) used were of chemical grade and purchased from Shenyang Sinopharm Reagent Co., Ltd. (Shenyang, China) without further purification.

### 2.2. The Intercalation Process of Sericite by Anionic–Cationic Surfactants CTAB and SDS

S_3_ was added to the suspension of DMSO and DI water (the volume ratio between dimethyl sulfoxide (DMSO) and DI water was 92:8) and stirred for 30 min. CTAB was added to the suspension in an amount 15 times the CEC of the modified sericite at 80 °C and stirred for 4 h. Following this, varying amounts of SDS (4 times, 10 times, 16 times, and 32 times the CEC of S_3_) were added to the above suspension. The mixture was stirred at various temperatures (60 °C, 70 °C, 80 °C, and 90 °C) and times (0.1 h, 1 h, 2 h, and 4 h). After washing with DI water 5 times, centrifugation at 4500 rpm for 4 min, and drying at 50 °C for 12 h, the CTAB-SDS co-intercalated sericite was obtained. The solely SDS intercalated sericite was prepared following the above procedure without the use of CTAB.

### 2.3. The Preparation Process of Sericite/Epoxy Composites

**Scheme 1:**
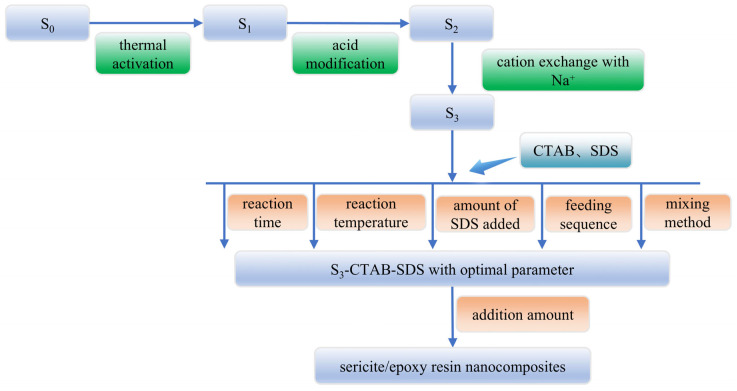
Flowchart of the modification, intercalation, and subsequent preparation of sericite/epoxy composites.

Various amounts of intercalated sericite were added to 20 mL acetone and stirred for 30 min to create a suspension. The suspension was then added into 100 g preheated E51 epoxy resin (preheated at 80 °C) and stirred for 2 h. Then the preheated curing agent 4,4’-diaminodiphenylmethane (DDM) (preheated at 80 °C) was added and stirred for 30 min. The molar ratio between the epoxy group and the amino group was 1:1. After curation at 80 °C for 2 h and 150 °C for 2 h, the final sericite/epoxy nanocomposites were obtained. The mass fractions of sericite to the total amount of epoxy resin and DDM were 0.1%, 0.5%, 1%, 1.5%, and 2%, respectively. To clarify the entire process, a flowchart (Scheme 1) is provided to illustrate the modification and intercalation of the sericite, as well as the subsequent preparation of sericite/epoxy composites.

### 2.4. Characterization Methods

**Figure 1:**
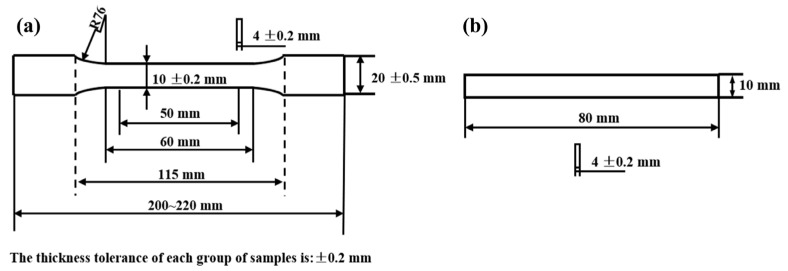
The shape of the sericite–epoxy resin composite for the test of (**a**) tensile strength and (**b**) bending strength.

X-ray diffraction (XRD) patterns of the samples were obtained from an Ultima IV X-ray diffractometer (PANalytical B.V., Eindhoven, The Netherlands) with 2θ range between 1° and 10° at 40 kV and 100 mA. The step size was 0.02° and the scanning speed was 0.5° (2θ)/min. Fourier transform infrared (FTIR) spectra were obtained using a Nicolet iS10 spectrometer (Thermo Fisher Scientific, Waltham, MA, USA) with a measuring range of 4000–450 cm^−1^ using a KBr pellet. The resolution was 4 cm^−1^ and the number of spectra acquired for the samples was 16. Scanning electron microscope (SEM) images were obtained by using a Hitachi S-4800 scanning electron microscope (Hitachi High-Technologies Corp., Tokyo, Japan). The accelerating voltage was set at 5 kV. SEM samples were prepared by using a toothpick to pick up a small amount of powder and pressing it onto the conductive tape, which had been previously attached to the sample holder. The powders were then pressed further to ensure firm contact with the tape. A Shimadzu DTG-60 simultaneous DTA-TG apparatus (TA instrument, Inc., New Castle, DE, USA) was used for thermal analysis of the samples with the temperature ranging from room temperature to 1000 °C at a heating rate of 10°/min. The samples were tested in a nitrogen atmosphere with N_2_ flow at the speed of 25 mL/min. The contact angles of the samples were measured by a Rame-Hart 200-F1 goniometer (Rame-hurt, Succasunna, NJ, USA) apparatus by adding water droplets on the surfaces of the pressed flat sheet of sericite to test the wettability. To estimate the mechanical properties of sericite/epoxy resin composites, tensile tests and bending tests were conducted with an electronic universal testing machine (WDW-300, Shimadzu, Kyoto, Japan) according to the GB/T 2567-2021 [29]. The samples for the test of bending strength were made in a rectangular shape and in a dumbbell shape, with the detailed information presented in Figure 1. To minimize the experimental errors, five samples were tested to obtain one average value.

## 3. Results and Discussion

### 3.1. The Effects of Co-Intercalation Conditions

#### 3.1.1. The Effects of Co-Intercalation Temperature

**Figure 2:**
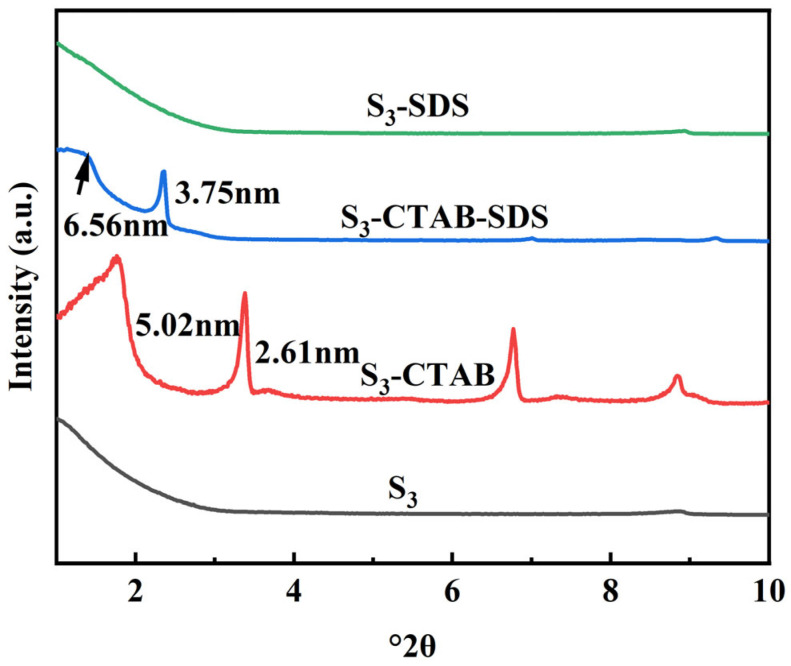
XRD patterns of the modified sericite (S_3_), CTAB-intercalated sericite (S_3_-CTAB), SDS intercalated sericite (S_3_-SDS), and CTAB-SDS co-intercalated sericite. (Reaction conditions: temperature: 80 °C, co-intercalation time: 4 h, SDS dosage: 16 times CEC.)

**Figure 3:**
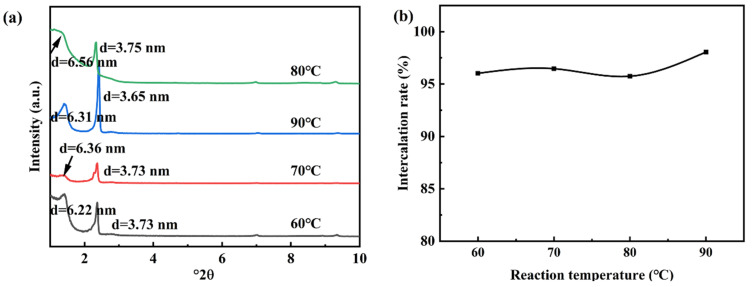
XRD patterns (**a**) and intercalation rate (**b**) of S_3_ with various temperatures. (Other reaction conditions are as follows: co-intercalation time: 4 h, SDS dosage: 16 times CEC).

As shown in Figure 2, anionic surfactant SDS could not be intercalated into the sericite interlayer space alone. This corresponded well with the previous reports [30]. Sericite can be intercalated, however, by cationic surfactant CTAB and anionic surfactant SDS, with the layer-to-layer distance increased to 6.56 nm. The relative parameters on the intercalation of sericite by cationic–anionic surfactants were explored systematically. As shown in Figure 3a, the largest increase in the d_001_ value (6.56 nm) occurred at 80 °C, with a relatively high intercalation rate of 95.7% (Figure 3b). As a consequence, 80 °C was selected as the optimal intercalation temperature, as the resulting intercalated sericite exhibited the largest interlayer spacing and a relatively high intercalation rate.

#### 3.1.2. The Effects of Co-Intercalation Time

**Figure 4:**
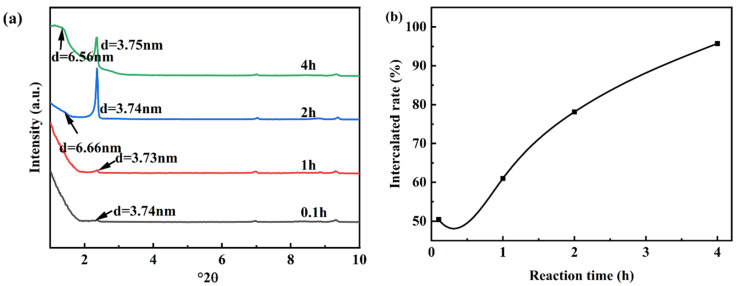
XRD patterns (**a**) and intercalation rate (**b**) of S_3_ with various times. (Other reaction conditions are as follows: co-intercalation temperature: 80 °C, SDS dosage: 16 times CEC).

Figure 4a showed that the interlayer space of sericite increased with intercalation time. At 0.1 h, the layer-to-layer distance of sericite was only 3.74 nm. After 2 h, however, it expanded significantly to 6.66 nm. This indicated that the intercalation is a gradual process; with shorter intercalation time, SDS barely penetrated the sericite layers. Over time, SDS gradually entered the interlayer, further expanding the spacing. The intercalation rate improved with reaction time, reaching 95.7% at 4 h (Figure 4b). As a consequence, 4 h was chosen as the optimal reaction time.

#### 3.1.3. The Effects of Co-Intercalation Agent Dosage

**Figure 5:**
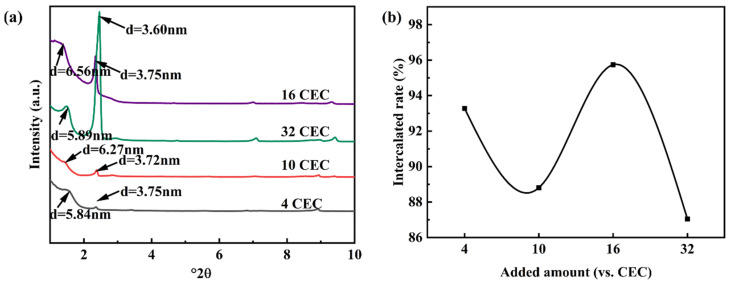
XRD patterns (**a**) and intercalation rate (**b**) of S_3_ with various co-intercalation agent dosage. (Other reaction conditions are as follows: co-intercalation temperature: 80 °C; co-intercalation time: 4 h).

Four SDS dosages were selected to study their effects on the intercalation of sericite: 4, 10, 16, and 32 times the CEC of sericite. At 16 times the CEC, the layer-to-layer distance of sericite reached 6.56 nm, which was the highest among these samples (Figure 5a). Similarly, the intercalation rate peaked at 95.7% at this dosage (Figure 5b). As a consequence, 16 times CEC was chosen as the optimal SDS dosage for intercalation.

#### 3.1.4. The Effects of Various Feeding Sequences and Mixing Methods

**Figure 6:**
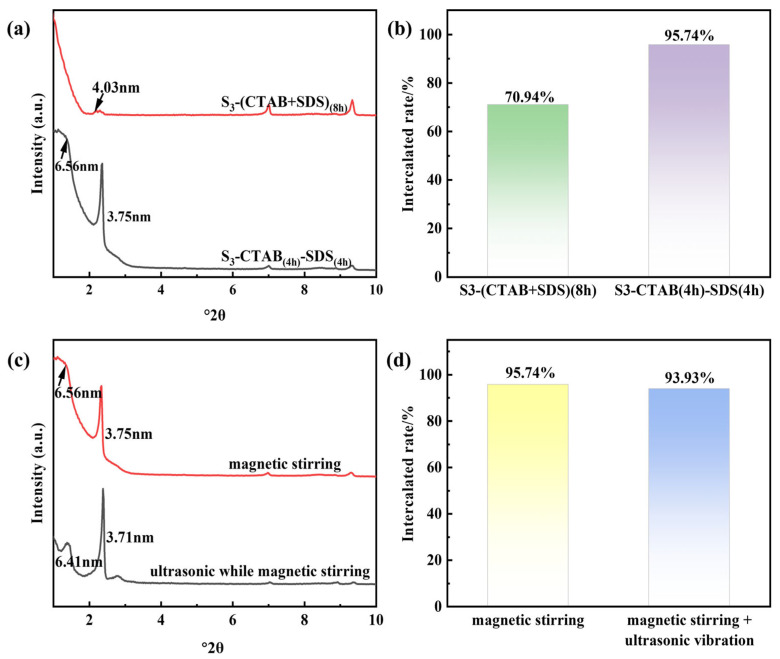
XRD patterns (**a**) and intercalation rate (**b**) of S_3_ with various feeding sequences. (S_3_-(CTAB+SDS) (8 h) means putting CTAB and SDS at the same time into the suspension and the intercalation time lasts for 8 h; S3-CTAB(4h)-SDS(4 h) means putting CTAB into the suspension first for 4 h and then putting SDS into the suspension for 4 h). XRD patterns (**c**) and intercalation rate (**d**) of S_3_ with various mixing methods.

In addition to the above-mentioned parameters, the effects of various feeding sequences were also researched. Figure 6a shows that sequential feeding (adding CTAB to the suspension first, followed by SDS) produced significantly better results than simultaneous feeding (adding CTAB and SDS at the same time). The layer-to-layer distance achieved by sequential feeding was 6.56 nm, much larger than the 4.03 nm obtained from simultaneous feeding. The corresponding intercalation rate for sequential feeding (95.7%) also exceeded that of simultaneous feeding (70.9%) (Figure 6b). This can be explained by the fact that in sequential feeding, CTAB first enlarged the interlayer space, facilitating the subsequent intercalation of SDS. In contrast, simultaneous feeding resulted in weaker ion-pair interactions between CTAB^+^ and SDS^−^, leading to lower interlayer space and intercalation rates. As a consequence, sequential feeding was selected as the optimal feeding sequence.

Ultrasonic vibration is known for improving the dispersion and exfoliation of materials [8], while stirring is more commonly used in the lab. To compare their effects, both methods were tested. Results showed stirring alone was more effective for sericite intercalation than the combination of stirring and ultrasonic vibration, both in terms of layer-to-layer distance (6.56 nm vs. 6.41 nm, Figure 6c) and intercalation rate (95.7% vs. 93.9%, Figure 6d). Because ultrasonic vibration had minimal impact in this experiment, stirring was chosen as the sole method for optimal results.

### 3.2. Characterization of the Obtained Anionic–Cationic Surfactants Co-Intercalated Sericite

**Figure 7:**
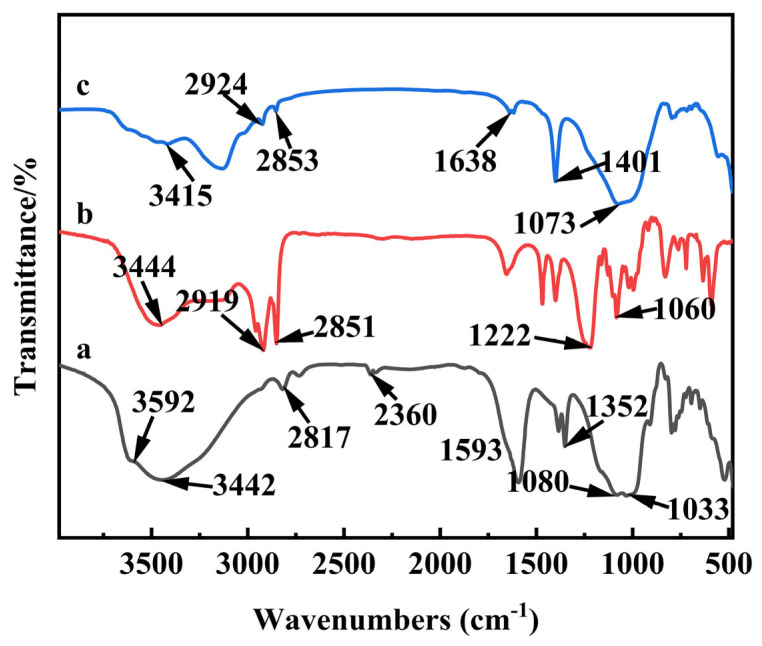
FTIR spectra of (a) sericite, (b) CTAB-SDS co-intercalated sericite, and (c) CTAB-intercalated sericite.

FTIR was further performed to prove the successful intercalation of anionic–cationic surfactants into sericite (Figure 7). In raw sericite (S_0_), the bands around 1080 cm^−1^ and 1033 cm^−1^ corresponded to the stretching vibrations of Si-O and Si-O-Si bonds [31]. The bands at 3592 cm^−1^ and 3442 cm^−1^ were attributed to the stretching vibrations of the Si(Al)-OH bond and the interlayer water molecules [32], respectively, while bands below 700 cm^−1^ corresponded to the bending vibrations of Si-oxygen-metal bond (such as Si-O-Al, Si-O-Mg, and Si-O-Fe). After CTAB intercalation, peaks at 2924 cm^−1^ and 2853 cm^−1^ can be assigned to the stretching bonds of CH_2_ in CTA^+^ [33], while the peak at 1401 cm^−1^ was attributed to the bending vibration of the N−H bond in CTA^+^ [34]. All of these proved the successful intercalation of CTA^+^ into the sericite interlayer. After the CTAB-SDS co-intercalation, a new peak at 1222 cm^−1^ emerged, which could be attributed to the vibration of the S=O bond in the SO_3_^2−^ group, while the peak at 1060 cm^−1^ was related to S-O stretching [35,36]. This confirmed the successful co-intercalation of CTAB and SDS into the interlayer space of sericite.

**Figure 8:**
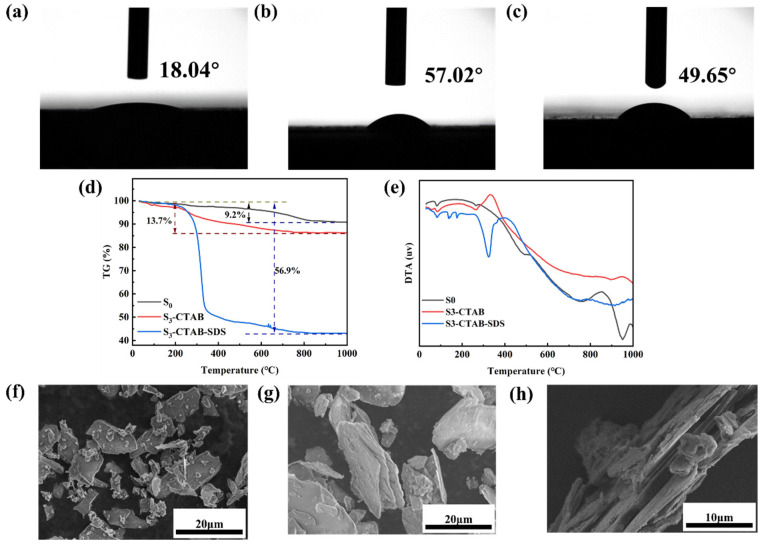
Contact angle tests of (**a**) sericite, (**b**) CTAB-intercalated sericite, and (**c**) CTAB-SDS co-intercalated sericite; (**d**) TG and (**e**) DTA curves of the samples; SEM images of (**f**) sericite, (**g**) CTAB-intercalated sericite, and (**h**) CTAB-SDS co-intercalated sericite.

Experimental measurements of contact angle (Figure 8a–c) revealed the sericite was originally hydrophilic before intercalation (18.04°). After CTAB intercalation, the contact angle increased to 57.02°, due to the incorporation of hydrophobic alkyl chain CTA^+^ into the interlayer. For CTAB-SDS co-intercalated sericite, the contact angle decreased to 49.65°, likely due to the introduction of SDS, which was more hydrophilic than CTAB. The less hydrophilic nature of the intercalated sericite suggested good adhesion with the polymer matrix. The total mass loss of sericite reached 9.2% (Figure 8d). DTA curves in Figure 8e showed an endothermic valley at 82 °C, which corresponded to the dehydration of water molecules absorbed in the interlayers, while the exothermic peak at 854 °C might have been caused by the structural failure due to the high temperature. In comparison, CTAB-intercalated sericite exhibited a distinct endothermic valley at 240 °C and an exothermic peak at 332 °C, consistent with results from the Zhang group [37]. The main endothermic valley of CTAB-SDS co-intercalated sericite appeared at 326.5 °C, which was significantly higher than that of CTAB-intercalated sericite. This can be verified by the corresponding TG tests, where CTAB-SDS co-intercalated sericite started to decompose at approximately 240 °C, while CTAB-intercalated sericite started to decompose at around 185 °C. This indicated that CTAB-SDS co-intercalated sericite had greater thermal stability. The mass loss of CTAB-SDS co-intercalated sericite was 56.9%, higher than that of CTAB-intercalated sericite (13.7%), further confirming successful co-intercalation by cationic–anionic surfactants.

The morphologies of the samples were also analyzed. The raw sericite exhibited smooth surfaces with sharp edges, indicating well-developed crystals, with an average size of 10–15 µm (Figure 8f). After CTAB intercalation, the interlayer space increased, confirming successful intercalation (Figure 8g). The surface of the sericite became less smooth, likely due to the modification process. In CTAB-SDS co-intercalated sericite, the interlayer space was also increased, with some layers partially exfoliated, providing additional evidence of successful intercalation of the anionic–cationic surfactants (Figure 8h).

### 3.3. Mechanism for Cationic–Anionic Surfactants Intercalated Sericite

**Figure 9:**
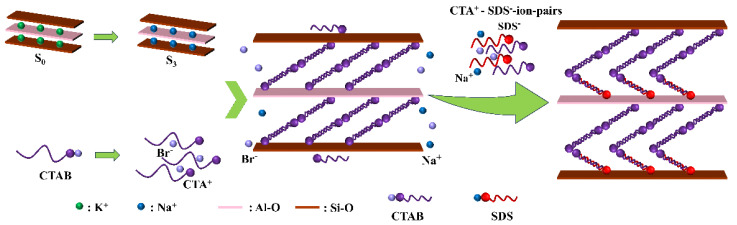
Intercalation mechanism of cationic–anionic surfactants into the interlayer of sericite.

The XRD pattern above clearly showed that anionic surfactant SDS could not be directly intercalated into sericite, meaning dodecanesulfonate ions (SDS^−^) could not enter the sericite layers on their own. However, the successful co-intercalation of the cationic–anionic surfactant pair, CTAB-SDS, was achieved. In this process, CTAB was first dissolved into the suspension, generating CTA^+^ and Br^−^. The generated CTA^+^ intercalated into sericite by ion exchange with Na^+^ [2], expanding the interlayer space and making the sericite hydrophobic, which facilitates subsequent SDS^−^ intercalation. When SDS was added, CTA^+^-SDS^−^ ion pairs could be formed, and entered the interlayer of sericite to further expand layer spacing. This mechanism is illustrated in Figure 9.

### 3.4. Mechanical Properties of Sericite/Epoxy Resin Composites

**Figure 10:**
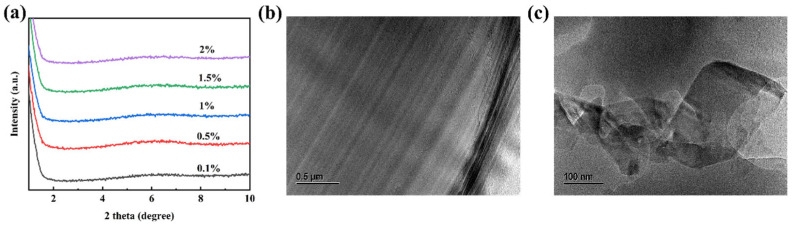
(**a**) XRD patterns of the sericite/epoxy resin with various amounts of anionic–cationic surfactants co-intercalated in sericite; (**b**,**c**) TEM images of sericite/epoxy nanocomposite with 0.5% sericite added.

A series of sericite/epoxy resin composites were fabricated with varying amounts of sericite, and the disappearance of all sericite-related peaks in the corresponding XRD patterns (Figure 10a) revealed the exfoliation of sericite in all composites, regardless of the sericite content (0.1% to 2%). This represented an improvement over previously reported results [15]. The distance between the lamellae of sericite in the TEM images exceeded 50 nm, indicating successful exfoliation (Figure 10b). In addition, exfoliated sericite nanosheets were observed, further confirming the exfoliation of sericite in the sericite/epoxy nanocomposite (Figure 10c).

**Figure 11:**
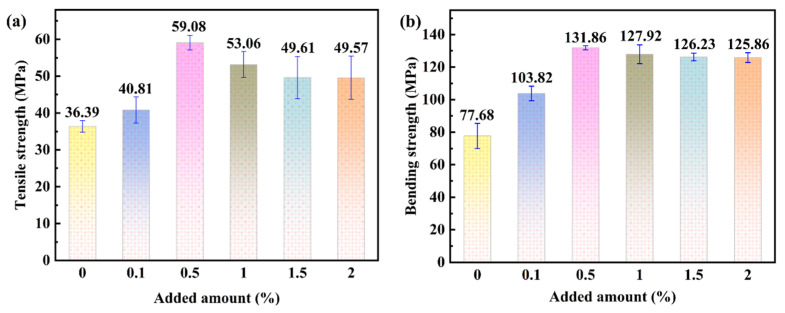
Mechanical properties ((**a**) tensile strength and (**b**) bending strength) of sericite/epoxy resin composite with various added amounts of sericite.

**Figure 12:**
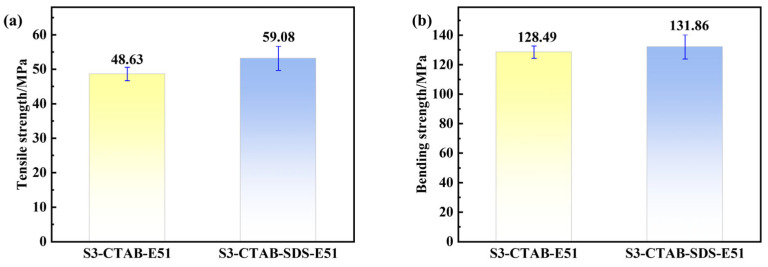
Comparison of the mechanical properties ((**a**) tensile strength and (**b**) bending strength) of sericite/epoxy resin composite with different sericites added (cationic CTAB-sericite and cationic–anionic CTAB-SDS-sericite). For each type of intercalated sericite, the addition amount was fixed at 0.5%.

Mechanical testing of the sericite/epoxy resin composites revealed that both bending strength and tensile strength were the best when 0.5% sericite was added (Figure 11a,b). Compared with pure epoxy resin, the bending and tensile strengths of sericite/epoxy resin composite with 0.5% sericite added increased by 72% and 62%, respectively, aligning well with previous findings [38,39]. Both the bending strength and tensile strength of the composites decreased as the sericite content exceeded 0.5%; however, they remained significantly higher than those of pure epoxy resin. This enhancement was attributed to the numerous interfaces formed between organo-sericite and the epoxy resin, which reduced shear zone formation and subsequent cracking, thereby improving tensile strength. In addition, both the bending strength and tensile strength of the sericite/epoxy resin composites with different sericites (one is cationic CTAB-sericite, the other is cationic–anionic CTAB-SDS-sericite) added were compared (Figure 12a,b). It can be seen that both the tensile strength and bending strength of CTAB-SDS-sericite are higher than those of CTAB-sericite, although the improvements are relatively modest. This can be attributed to the better compatibility of SDS with epoxy resin compared to CTAB.

## 4. Conclusions

This study demonstrated that sericite could not be directly intercalated with anionic surfactants into the interlayer space, while successful intercalation occurred with cationic–anionic CTAB-SDS surfactants. The relative parameters, such as intercalation time, intercalation temperature, and intercalation agent dosage were optimized. After co-intercalation, the layer-to-layer distance was increased to 6.56 nm, with the intercalation rate reaching 95.74%. The co-intercalated sericite exhibited greater hydrophobicity than raw sericite and improved thermal stability compared to CTAB-intercalated sericite. The intercalated sericite was subsequently incorporated into epoxy resin to form sericite/epoxy composites, which exhibited superior mechanical properties, with significant improvements in tensile and bending strengths compared to both pure epoxy resin and CTAB-intercalated sericite. These results provide valuable insights for the development of novel organo-sericite materials with enhanced mechanical properties and thermal stability, suitable for applications in polymer composites, oil well drilling, and paints.

## Data Availability

The data presented in this study are available on request from the corresponding authors.
